# Circulating cell-death–associated biomarkers and inflammatory severity in Hymenoptera sting envenomation: a single-center retrospective study

**DOI:** 10.3389/fcell.2026.1970146

**Published:** 2026-10-14

**Authors:** Pan Bian, Yuhua Wang, Shaohui Zhang, Yang Gui, Zhenya Shen

**Affiliations:** Department of Emergency Medicine, The First Affiliated Hospital of Henan University of Science and Technology, Luoyang, Henan, China

**Keywords:** GSDMD, Hymenoptera envenomation, IL-18, IL-1β, retrospective cohort, RIPK3, severity, wasp sting

## Abstract

**Background:**

Severe Hymenoptera sting envenomation can cause systemic inflammation, shock, and multi-organ injury. Gasdermin D cleavage and RIPK3 phosphorylation are associated with inflammatory cell-death pathways, but circulating measurements cannot by themselves establish a specific cellular death mechanism. We therefore evaluated the clinical associations of serum GSDMD-p30, p-RIPK3, IL-1β, and IL-18 with illness severity in patients with Hymenoptera sting envenomation.

**Methods:**

We conducted a single-center retrospective study of 142 adults admitted between January 2023 and December 2025. Clinical severity was categorized at admission using a four-level institution-specific scheme: Grade I, local/minimal systemic manifestations without organ dysfunction; Grade II, systemic allergic manifestations without organ dysfunction; Grade III, single-organ failure; and Grade IV, multi-organ failure and/or anaphylactic shock. Archived serum was available for 98 patients (Grade I/II/III/IV: 30/33/22/13). Serum GSDMD-p30, p-RIPK3, IL-1β, and IL-18 were measured by ELISA-based assays. Associations with severity grade and SOFA score and exploratory same-cohort ROC performance for Grade III-IV disease were assessed.

**Results:**

The cohort included 42 Grade I, 48 Grade II, 34 Grade III, and 18 Grade IV patients. Sting count, time to hospital presentation, inflammatory indices, organ-injury markers, SOFA score, ICU admission, and length of stay increased with clinical severity. In the serum subset, mean GSDMD-p30 increased from 0.82 ± 0.21 ng/mL in Grade I to 9.43 ± 2.18 ng/mL in Grade IV, and p-RIPK3 increased from 0.64 ± 0.18 to 7.84 ± 1.87 ng/mL (global p < 0.001 for both). IL-1β and IL-18 showed similar severity-associated increases. Correlations with SOFA score were ρ = 0.814 for GSDMD-p30, ρ = 0.783 for p-RIPK3, ρ = 0.791 for IL-18, and ρ = 0.762 for IL-1β (all p < 0.001). Apparent AUCs for Grade III-IV disease were 0.861, 0.843, 0.834, and 0.812, respectively.

**Conclusion:**

Circulating GSDMD-p30, p-RIPK3, IL-1β, and IL-18 were associated with clinical severity and organ-failure burden in this retrospective cohort. These findings support further evaluation of these proteins as candidate severity-associated biomarkers, but they do not demonstrate PANoptosis, establish cell-specific origin, or validate a clinical triage tool. Prospective multicenter studies with standardized assays and adjusted analyses are required.

## 1 Introduction

Hymenoptera stings produce a spectrum of clinical presentations ranging from local reactions and IgE-mediated anaphylaxis to direct toxic envenomation with rhabdomyolysis, hemolysis, acute kidney injury, hepatic injury, shock, and multi-organ dysfunction (Schmidt, 2018; Xie et al., 2013; Dhanapriya et al., 2016; Vikrant and Parashar, 2017; Gong et al., 2019; Golden, 2015). The risk of toxic organ injury generally increases with venom burden, and delayed presentation may further complicate clinical management (Schmidt, 2018; Xie et al., 2013; Vikrant and Parashar, 2017; Gong et al., 2019). In large Chinese cohorts of severe wasp stings, toxic organ injury has accounted for a substantial proportion of serious outcomes, emphasizing the need for early severity assessment (Xie et al., 2013; Vikrant and Parashar, 2017).

Hymenoptera venoms contain biologically active peptides and enzymes capable of membrane injury and inflammatory activation (Habermann, 1972; Lee and Bae, 2016; Pucca et al., 2019). Among inflammatory cell-death pathways, cleavage of gasdermin D (GSDMD) generates a pore-forming N-terminal fragment that is a key executor of pyroptosis, whereas receptor-interacting protein kinase 3 (RIPK3) participates in necroptotic signaling (Shi et al., 2015; Linkermann and Green, 2014; Ding et al., 2016; Kayagaki et al., 2015; Newton et al., 2019). NLRP3 inflammasome activation can promote maturation of IL-1β and IL-18 (Swanson et al., 2019). PANoptosis has been proposed as a coordinated inflammatory cell-death program integrating pyroptotic, apoptotic, and necroptotic machinery (Pandian and Kanneganti, 2022; Christgen et al., 2022). However, demonstration of PANoptosis requires functional evidence of coordinated pathway activation and cannot be inferred from serum proteins alone.

In this clinical retrospective study, we assessed whether serum GSDMD-p30, p-RIPK3, IL-1β, and IL-18 were associated with clinical severity and SOFA score in patients hospitalized after Hymenoptera stings. These measurements are interpreted as circulating severity-associated biomarkers rather than evidence of a specific monocyte population or a demonstrated PANoptotic mechanism.

## 2 Methods

### 2.1 Study design and patient selection

We conducted a retrospective study of adults admitted to the Emergency Department or ICU of The First Affiliated Hospital, College of Clinical Medicine of Henan University of Science and Technology between January 2023 and December 2025. Inclusion criteria were age ≥18 years; a documented Hymenoptera sting exposure recorded as Vespa velutina, Vespa mandarinia, or Vespa basalis; and available clinical records including sting count, time to hospital presentation, laboratory data, and in-hospital outcome. Exclusion criteria were known immunosuppression or autoimmune disease, anticoagulation therapy, a prior history of severe anaphylaxis judged to confound the institutional severity classification, or incomplete medical records. Of 196 screened patients, 142 met the eligibility criteria.

The study was approved by the Ethics Committee of the First Affiliated Hospital of Henan University of Science and Technology (No. K-2025-B137), with waiver of individual informed consent for retrospective chart review.

### 2.2 Clinical severity classification

Clinical severity was recorded at admission using a pre-specified four-level institutional classification: Grade I, local reaction or minimal systemic manifestations without generalized allergic features or organ dysfunction; Grade II, systemic allergic manifestations without organ dysfunction; Grade III, single-organ failure attributable to envenomation; and Grade IV, multi-organ failure and/or anaphylactic shock. The resulting distribution was Grade I, n = 42; Grade II, n = 48; Grade III, n = 34; and Grade IV, n = 18. Biomarker measurements from archived serum were not used to assign severity grade.

This four-level scheme is institution-specific and has not undergone independent external validation. It is not intended to replace established anaphylaxis definitions or severity frameworks such as those described in international allergy guidance and published clinical grading systems (Golden, 2015; Cardona et al., 2020; Sturm et al., 2018; Bilò et al., 2016; Brown, 2004). We therefore use the grades only as an operational retrospective stratification of this cohort and avoid treating them as a universally validated staging system.

### 2.3 Clinical variables and outcomes

Demographic and clinical variables extracted from the records included age, sex, sting count, time from sting to hospital presentation, white blood cell count (WBC), C-reactive protein (CRP), alanine aminotransferase (ALT), aspartate aminotransferase (AST), creatinine, lactate dehydrogenase (LDH), SOFA score, ICU admission, in-hospital mortality, and length of hospital stay. SOFA was used as a measure of organ-dysfunction burden (Singer et al., 2016). The primary analyses examined biomarker differences across the four institutional severity grades and correlations with SOFA score. Grade III-IV was used only as an exploratory dichotomous severe-disease endpoint for ROC analysis.

### 2.4 Serum biomarker measurement

Archived serum stored at −80 °C was available for 98 of 142 patients (Grade I, n = 30; Grade II, n = 33; Grade III, n = 22; Grade IV, n = 13). Serum IL-1β and IL-18 were quantified by ELISA (R&D Systems DY201 and DY318-05, respectively). GSDMD-p30, representing the cleaved GSDMD fragment, and phospho-RIPK3 (p-RIPK3 Thr231/Ser232) were measured using custom sandwich ELISA procedures with matched antibody reagents (Abcam ab219800/ab227170 for GSDMD; Cell Signaling Technology #91702 for p-RIPK3). All measurements were performed in duplicate, and the reported inter-assay coefficient of variation was <10%. These custom assays were used for research purposes and were not used for clinical decision-making.

cBecause the custom GSDMD-p30 and p-RIPK3 assays are not established clinical diagnostic tests, the present study does not claim full analytical validation. Additional assay characteristics requested for clinical translation—including lower limits of detection, dynamic range, intra-assay precision, antibody-specificity testing, freeze-thaw stability, and blinded inter-operator reproducibility—require prospective standardized evaluation.

### 2.5 Statistical analysis

Continuous variables are presented as median [interquartile range, IQR] or mean ± SD as reported in the clinical dataset, and categorical variables as n (%). Variables summarized as median [IQR] were compared across the four severity grades using the Kruskal–Wallis test. Variables summarized as mean ± SD were compared using Welch one-way ANOVA to reduce sensitivity to unequal group variances. Categorical variables were compared using Pearson chi-square tests when expected cell counts were adequate and the Fisher-Freeman-Halton exact test for sparse 4 × 2 tables. Spearman rank correlation (ρ) was used for associations between serum biomarkers and SOFA score. For the four prespecified biomarker comparisons across severity grades and the four prespecified biomarker-SOFA correlations, a Bonferroni-adjusted significance threshold of 0.0125 was used; all reported biomarker results remained significant at p < 0.001. ROC curves were used only to describe apparent, same-cohort discrimination of individual biomarkers for Grade III-IV disease; no inferential comparison between AUCs, composite score, treatment threshold, or externally validated prediction model is presented in this study.

To partially assess selection related to archived-serum availability, the four-grade severity distribution was compared between patients with and without archived serum. Because sting number and time to hospital presentation differed materially across severity groups and other potential confounders may also affect outcome, the biomarker analyses are interpreted as unadjusted exploratory associations rather than independent causal or prognostic effects. Comparisons in Tables 1, 2 are descriptive and exploratory; their p values were not adjusted for multiplicity. No *post hoc* pairwise tests were performed, and all reported across-grade p values are global omnibus tests. All tests were two-sided, with p < 0.05 considered statistically significant unless otherwise specified.

**TABLE 1:** Demographic, exposure, and clinical outcome characteristics by institutional severity grade.

Characteristic	Grade I (n = 42)	Grade II (n = 48)	Grade III (n = 34)	Grade IV (n = 18)	p-value
Age, years (median [IQR])	51 [40–62]	53 [42–65]	54 [43–66]	52 [38–63]	0.812
Sex, male, n (%)	24 (57.1)	29 (60.4)	21 (61.8)	10 (55.6)	0.961
Sting count (median [IQR])	11 [7–18]	32 [22–41]	59 [45–76]	105 [85–168]	<0.001
Time to hospital, h (median [IQR])	1.4 [0.8–2.2]	1.6 [0.9–2.5]	2.1 [1.3–3.4]	2.8 [1.5–4.1]	0.003
Archived serum available, n (%)	30 (71.4)	33 (68.8)	22 (64.7)	13 (72.2)	0.919
ICU admission, n (%)	0 (0)	2 (4.2)	14 (41.2)	10 (55.6)	<0.001
In-hospital mortality, n (%)	0 (0)	0 (0)	1 (2.9)	2 (11.1)	0.013
Hospital stay, days (median [IQR])	2 [1–3]	4 [3–6]	11 [8–15]	18 [13–26]	<0.001

IQR, interquartile range; ICU, intensive care unit. Global p values: age, sting count, time to hospital, and hospital stay, Kruskal–Wallis test; sex and archived-serum availability, Pearson chi-square test; ICU admission and in-hospital mortality, Fisher-Freeman-Halton exact test because of sparse cells. These descriptive p values are exploratory and were not adjusted for multiplicity.

**TABLE 2:** Inflammatory, organ-injury, and organ-dysfunction indices by institutional severity grade.

Characteristic	Grade I (n = 42)	Grade II (n = 48)	Grade III (n = 34)	Grade IV (n = 18)	p-value
WBC (×10^9^/L)	9.2 ± 2.1	11.8 ± 2.8	17.4 ± 4.2	22.6 ± 5.8	<0.001
CRP (mg/L)	18.3 ± 6.4	42.1 ± 12.3	98.7 ± 28.4	187.4 ± 52.1	<0.001
ALT (U/L)	28 ± 11	74 ± 22	142 ± 48	187 ± 54	<0.001
AST (U/L)	31 ± 13	88 ± 31	198 ± 67	312 ± 89	<0.001
Creatinine (μmol/L)	74 ± 18	112 ± 34	287 ± 98	387 ± 142	<0.001
LDH (U/L)	234 ± 62	482 ± 118	1,124 ± 312	1,842 ± 587	<0.001
SOFA score (median [IQR])	0 [0–1]	2 [1–3]	6 [5–8]	11 [9–14]	<0.001

Values are mean ± SD, unless otherwise stated. Global p values for WBC, CRP, ALT, AST, creatinine, and LDH are from Welch one-way ANOVA; the SOFA p value is from the Kruskal–Wallis test. These descriptive p values are exploratory and were not adjusted for multiplicity. WBC, white blood cell count; CRP, C-reactive protein; ALT, alanine aminotransferase; AST, aspartate aminotransferase; LDH, lactate dehydrogenase; SOFA, sequential organ failure assessment; IQR, interquartile range.

## 3 Results

### 3.1 Study population and clinical severity distribution

Between January 2023 and December 2025, 196 patients were screened. Fifty-four were excluded (17 with incomplete records, 14 with a prior history of severe anaphylaxis, 12 with immunosuppression, and 11 aged <18 years), leaving 142 adults for the clinical analysis (Figure 1). The cohort comprised 42 Grade I, 48 Grade II, 34 Grade III, and 18 Grade IV patients. Median age was 52.4 years [IQR 41–64], and 84 patients (59.2%) were male.

**FIGURE 1:**
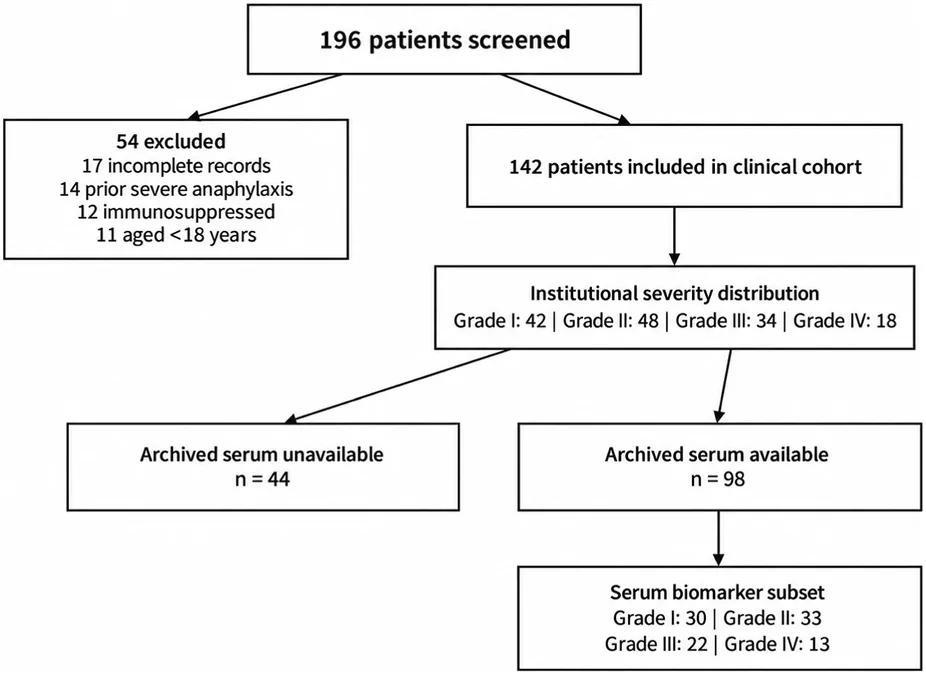
Study flow and formation of the archived-serum biomarker subset. The diagram shows screening, exclusions, the 142-patient clinical cohort, institutional severity distribution, and availability of archived serum for 98 patients.

Demographic characteristics were similar across severity grades, whereas sting burden, time to hospital presentation, ICU admission, in-hospital mortality, and length of stay showed clinically important gradients (Table 1). Archived serum was available in 98 patients; the proportion with available serum was similar across grades (global Pearson chi-square p = 0.919).

### 3.2 Exposure burden and organ-injury profile

Median sting count increased from 11 [7 -18] in Grade I to 105 [85 -168] in Grade IV (global p < 0.001; Figure 2). Time to hospital presentation also increased with severity (p = 0.003). In parallel, WBC, CRP, ALT, AST, creatinine, LDH, and SOFA score increased across the four institutional severity categories (all global p < 0.001; Table 2). These gradients indicate that exposure burden and presentation delay are important potential confounders of biomarker-severity associations.

**FIGURE 2:**
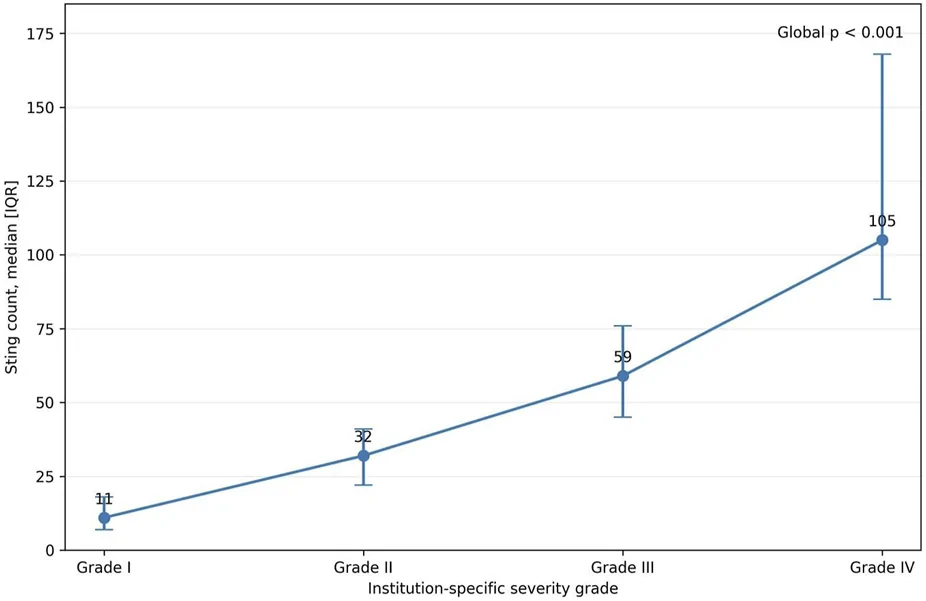
Sting burden across institutional severity grades. Points show the reported median sting count and vertical ranges show the interquartile range. The global p value is from the Kruskal–Wallis test; no pairwise comparisons are shown. The figure summarizes group-level data and does not display individual-patient observations.

### 3.3 Archived-serum subset and severity-associated biomarker levels

Archived serum was available for 98 patients (Grade I, n = 30; Grade II, n = 33; Grade III, n = 22; Grade IV, n = 13). The four-grade distribution did not differ significantly between patients with and without archived serum (Pearson chi-square p = 0.919), and Grade III-IV disease occurred in 35.7% (35/98) of the serum-available group versus 38.6% (17/44) of the serum-unavailable group. All four measured biomarkers were progressively higher across severity grades (Welch ANOVA global p < 0.001 for each; Table 3).

**TABLE 3:** Serum biomarker concentrations by institutional severity grade in the archived-serum subset.

Biomarker	Grade I (n = 30)	Grade II (n = 33)	Grade III (n = 22)	Grade IV (n = 13)	p-value
GSDMD-p30 (ng/mL)	0.82 ± 0.21	2.14 ± 0.48	5.87 ± 1.24	9.43 ± 2.18	<0.001
p-RIPK3 (ng/mL)	0.64 ± 0.18	1.73 ± 0.41	4.28 ± 0.98	7.84 ± 1.87	<0.001
IL-1β (pg/mL)	11.8 ± 2.9	38.4 ± 9.2	94.7 ± 18.3	241.6 ± 46.8	<0.001
IL-18 (pg/mL)	39.2 ± 7.8	88.6 ± 16.4	198.4 ± 34.7	312.8 ± 58.3	<0.001

Values are mean ± SD. Global p values are from Welch one-way ANOVA. for the four prespecified biomarker comparisons, the Bonferroni-adjusted significance threshold was 0.0125; all four remained significant at p < 0.001. GSDMD, gasdermin D; p-RIPK3, phosphorylated receptor-interacting protein kinase 3; IL, interleukin.

For visual comparison across assays reported in different concentration units, Figure 3 expresses each biomarker as fold change relative to its Grade I mean. This normalization is descriptive only; absolute concentrations and statistical comparisons are reported in Table 3.

**FIGURE 3:**
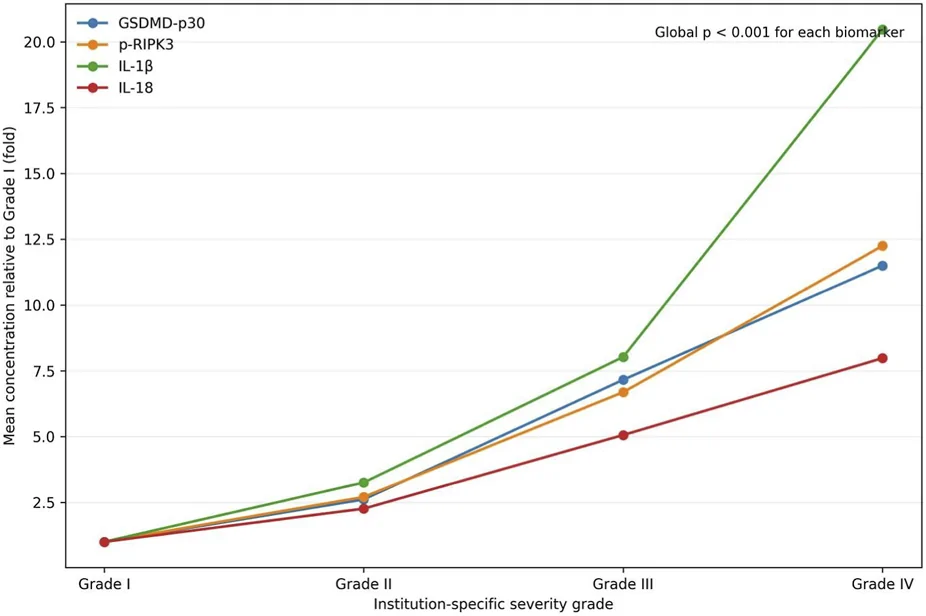
Severity-associated gradients in circulating biomarkers. Each line shows the group mean normalized to the corresponding Grade I mean (Grade I = 1) to permit visual comparison across biomarkers measured in different units. Absolute concentrations are provided in Table 3. Global p values are from Welch one-way ANOVA; all four biomarkers remained significant at the Bonferroni-adjusted threshold of 0.0125.

### 3.4 Association with SOFA score and exploratory discrimination

Serum GSDMD-p30 correlated with SOFA score (Spearman ρ = 0.814, p < 0.001), followed by IL-18 (ρ = 0.791, p < 0.001), p-RIPK3 (ρ = 0.783, p < 0.001), and IL-1β (ρ = 0.762, p < 0.001). All four associations remained significant under the prespecified Bonferroni threshold of 0.0125. Because sting burden and presentation delay differed across severity grades, these correlations are interpreted as unadjusted severity associations rather than independent effects.

In the 98-patient serum subset, 35 patients had Grade III-IV disease. Same-cohort ROC analysis yielded apparent AUCs of 0.861 for GSDMD-p30, 0.843 for p-RIPK3, 0.834 for IL-18, 0.812 for IL-1β, and 0.807 for LDH (Table 4; Figure 4). These estimates represent discovery-cohort discrimination only; no independent validation, optimism correction, composite score, or clinically actionable cutoff is claimed.

**TABLE 4:** Exploratory apparent discrimination of individual biomarkers for Grade III-IV disease in the archived-serum subset (n = 98).

Biomarker	AUC (95% CI)
Serum GSDMD-p30	0.861 (0.782–0.940)
Serum p-RIPK3	0.843 (0.761–0.925)
Serum IL-18	0.834 (0.746–0.922)
Serum IL-1β	0.812 (0.721–0.903)
Serum LDH	0.807 (0.714–0.900)

AUC, area under the receiver operating characteristic curve; CI, confidence interval. Estimates are apparent same-cohort performance and are not externally validated clinical thresholds. No formal statistical comparison among AUCs was performed.

**FIGURE 4:**
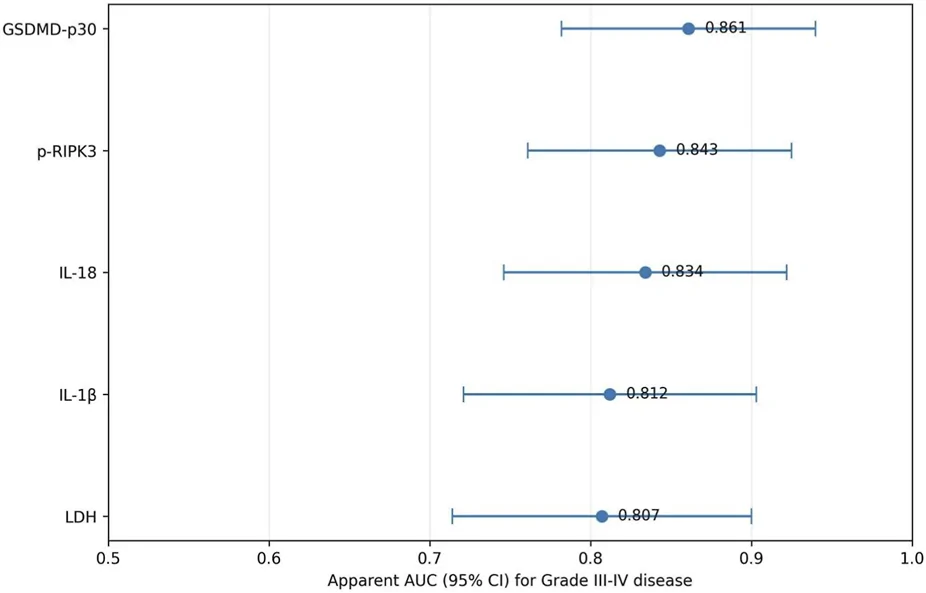
Apparent same-cohort discrimination for Grade III-IV disease. Points show AUC estimates and horizontal error bars show 95% confidence intervals. The x-axis begins at AUC = 0.50, corresponding to chance-level discrimination.

## 4 Discussion

In this single-center retrospective cohort, serum GSDMD-p30, p-RIPK3, IL-1β, and IL-18 increased with the institution-specific clinical severity grade and correlated with SOFA score. The present study therefore supports a clinical association between these circulating proteins and organ-dysfunction burden after Hymenoptera envenomation. It does not provide single-cell evidence, identify a novel monocyte population, or demonstrate that PANoptosis occurs in a specific circulating cell population.

The clinical gradient observed across severity groups is consistent with established literature showing that toxic Hymenoptera envenomation can produce acute kidney injury, hepatic injury, rhabdomyolysis, shock, and multi-organ dysfunction, particularly after multiple stings (Schmidt, 2018; Xie et al., 2013; Dhanapriya et al., 2016; Vikrant and Parashar, 2017; Gong et al., 2019). In our cohort, sting count and time to hospital presentation differed substantially across severity grades. These variables are clinically important potential confounders: patients with larger venom exposure or delayed presentation may simultaneously have higher circulating injury/inflammation markers and worse outcomes. Accordingly, the present study does not describe the biomarkers as independent predictors.

The biological interpretation of GSDMD-p30 and p-RIPK3 also requires restraint. GSDMD cleavage and membrane-pore formation are established molecular events in pyroptosis (Shi et al., 2015; Ding et al., 2016; Kayagaki et al., 2015), whereas RIPK3 is central to necroptotic signaling and interacts with broader cell-death pathway regulation (Linkermann and Green, 2014; Newton et al., 2019). IL-1β and IL-18 are linked to inflammasome activation (Swanson et al., 2019). Nevertheless, serum detection cannot determine the cellular source of these proteins, cannot show simultaneous execution of pyroptosis, apoptosis, and necroptosis in the same cell, and cannot establish PANoptosome formation. Thus, although PANoptosis provides a relevant conceptual framework for inflammatory cell death (Pandian and Kanneganti, 2022; Christgen et al., 2022), our clinical data should be viewed as indirect and hypothesis-generating rather than mechanistic proof.

The ROC analysis likewise requires cautious interpretation. The AUC estimates were obtained in the same 98-patient subset in which the biomarkers were studied, with only 35 Grade III-IV cases. No independent validation cohort, train-test split, bootstrap optimism correction, or repeated cross-validation was available in the original clinical analysis. We therefore report only apparent individual-marker discrimination. These values should not be used for clinical decision-making without prospective validation.

Several limitations remain. First, the retrospective, single-center design limits causal inference and generalizability. Second, the four-grade severity scheme is institution-specific and not externally validated; its use should therefore be interpreted as cohort stratification rather than a universal staging system. Third, archived serum was unavailable for 44 patients. Although the severity-grade distribution was similar between patients with and without serum, selection bias based on other characteristics cannot be excluded. Fourth, important confounding by sting burden, time to presentation, age, comorbidity, venom species, and pre-hospital treatment may remain; a fully adjusted patient-level analysis is required before independent biomarker effects can be claimed. Fifth, GSDMD-p30 and p-RIPK3 were measured by research custom assays for which complete clinical-grade analytical validation was not established in this retrospective study. Finally, serum biomarkers are not cell-specific and cannot demonstrate PANoptosis or pathway interdependence. These limitations define the study as exploratory and hypothesis-generating.

## 5 Conclusion

In this retrospective cohort of 142 adults with Hymenoptera sting envenomation, serum GSDMD-p30, p-RIPK3, IL-1β, and IL-18 were higher in more severe clinical groups and correlated with SOFA score in the 98 patients with archived serum. These proteins may represent candidate severity-associated biomarkers, but the present data do not establish PANoptosis, cell-specific origin, independent prognostic value, or a validated triage threshold. Prospective multicenter studies with standardized assay validation, complete covariate adjustment, and independent model validation are required before clinical implementation.

## Data Availability

The data supporting the findings of this study are available from the corresponding author upon reasonable request.
